# *“The balloon was just the kick start, I had to do the rest myself”*: Adolescents living with severe obesity experiences of an intra-gastric balloon alongside a lifestyle support programme

**DOI:** 10.1186/s12887-021-02902-x

**Published:** 2021-10-01

**Authors:** Lindsey J. Reece, Paul Bissell, Pooja Sachdev, Neil Wright, Seema Mihrshahi, Robert J. Copeland

**Affiliations:** 1 SPRINTER Research Group, Prevention Research Collaboration, School of Public Health, University of Sydney, Sydney, NSW Australia; 2 Advanced Wellbeing Research Centre, National Centre for Sport and Exercise Medicine, Sheffield Hallam University, Sheffield, UK; 3 School of Human and Health Sciences, Department of Allied Health Professions, Sport and Exercise, University of Huddersfield, Huddersfield, UK; 4 Nottingham University NHS Trust, Sheffield, UK; 5 Sheffield Children’s Hospital, Sheffield, UK; 6 Department of Healthy Systems and Populations, Faculty of Medicine, Health and Human Sciences, Macquarie University, Sydney, NSW Australia

**Keywords:** Adolescent, Severe obesity, Treatment

## Abstract

**Background:**

Few treatments exist for adolescents living with severe obesity. This qualitative study explored the experiences of severely obese adolescents and their families who participated in the BOB study.

**Methods:**

Twelve adolescents (5 males;7 females; mean age 15 years; BMI > 3.5 s.d; puberty stage 4 +) who were engaged with the research study BOB (a non-randomised, pilot novel obesity treatment programme that involved the insertion of an intra-gastric balloon coupled with a family lifestyle behavioural support programme). Adolescents attended weekly lifestyle sessions before, during and post balloon insertion. All participants were interviewed at 3 months, (halfway through intra-gastric balloon insertion) and at 12 months follow-up (6 months post intra-gastric balloon removal, 3 months post lifestyle intervention).

**Results:**

All BOB participants had exhausted all treatment options deeming this study their final option. Many alluded to feelings of desperation and referred to a sense of hope that this intervention would be effective. Family involvement and attendance within the structured sessions differed significantly. Adolescents and parents perceived support from the research study ceased when the intra-gastric balloon was removed at 6-months despite attendance post balloon removal being poor. All participants emphasised a need for further support longer term with the integration of the family a critical factor.

**Conclusions:**

Further research is needed to explore the specific role families play within treatment to optimise health and wellbeing outcomes. Adolescents perspectives should be integrated within treatment to inform and improve the effectiveness of future treatment programmes for severely obese adolescents and their families.

## Introduction

Children living with overweight and obesity are a global public health concern. The complexity of the obesity crisis means there are no effective treatments appropriate for all populations, resulting in treatment options typically determined by a child’s age and degree of obesity [1]. Common treatment options range from non-invasive lifestyle behavioural modification programs which focus on physical activity and dietary behaviours, through to more intensive options including medication and bariatric surgery [1].

Severe adolescent obesity is associated with high morbidity and mortality [2]. Whilst, a growing body of evidence advocates for a multicomponent, family-focused approach to treatment [3, 4], evidence from drug and surgical interventions remain limited with concerns about their safety and use in youth populations [1]. Research on the effects of child and adolescent obesity treatment has shown that short-term weight loss is often not sustained long-term [5]. Successful treatment is typically associated with sustained weight loss outcomes, but less is known about the broader psychosocial context surrounding participants including the experiences of individuals seeking to lose weight [4]. There is limited evidence regarding which individuals are most likely to benefit from a given treatment [6]. A better understanding of the predictors of change and the mediating factors influencing successful or unsuccessful behavioural change [7] could facilitate the effective tailoring of treatment to adolescent participant characteristics [6]. Treatment is difficult with adherence to weight loss programmes amongst adolescents as low as 15-20%, which generally reduces further as Body Mass Index (BMI) increases [8].

This study, BOB, a novel non-randomised pilot study targeting 12 adolescents with severe obesity with an average of 15 years old and a BMI > 3.5 s.d, aimed to reduce body weight and BMI Z score at 12 months, following adherence to the lifestyle programme for 9 months and an intra-gastric balloon inserted for 6 months [9, 10]. This study, the first to specifically tailor a treatment option for severely obese adolescents and their families at the time, demonstrated the potential to produce short term reductions in BMI score and improvements in physical fitness [10], both known to have beneficial impacts on health and wellbeing later in life.

When severely obese adolescents are engaged in treatment, little is known about their experiences in relation to such lifestyle and physical activity interventions [11, 12]. Qualitative research, which focuses on the individuals’ experiences during and after participation in obesity treatment, provides a technique to explore the acceptability of obesity treatment interventions along with the exploration of their impact on the lives of the participants [13]. A better understanding of the predictors of change and the mediating factors influencing successful or unsuccessful behavioural change [7] could facilitate the effective tailoring of future treatment’s to adolescent participant characteristics [6].

This qualitative study aimed to explore the experiences of all the adolescents and their families who participated in the BOB study - a novel obesity treatment programme that involved the insertion of an intra-gastric balloon coupled with a family lifestyle behavioural support programme.

## Methods

Ethics approval for the qualitative component of the BOB study was covered under the National Health Service (NHS) and written consent granted for the BOB study.

### Participants

All participants enrolled on the BOB pilot study (*N* = 12) were invited to be interviewed as part of their weekly attendance at the lifestyle programme at the university research facility, at 3 months, (half way through having the intra-gastric balloon in) and at 12 months follow-up (6 months post intra-gastric balloon removal, 3 months post lifestyle intervention). Interviews were held at 3 months (*n* = 11) and at 12 months (*n* = 9). Only one participant chose to be interviewed alone, all others had family or friends with them.

### Interview method

Interviews lasted between 35 and 60 min. An interview guide containing eight open questions was used. The open questions provided a framework, where topics were identified from previous reviews of childhood and adolescent obesity treatment interventions along with consultations with key stakeholders involved in obesity management [5, 14]. Key topics within the guide included questions focused on the expectations of the intervention, successful and unsuccessful changes, unanticipated setbacks, acceptability of the programme and future goals. Adhering to this guide for all interviews ensured consistency, whilst remaining flexible enough to adapt to the emerging accounts of the young people. The script, along with flip chart paper, was used to support reflection and interaction, offering additional prompts to help the young people think carefully about their experiences. Appreciating that these adolescents had very low self-esteem and were not necessarily confident about talking frankly and openly within an interview situation, the session was interactive allowing for responses to be written on coloured post it notes with creative artwork encouraged. To initiate conversation, all participants and their families were asked to introduce themselves with a general discussion around hobbies and interests. The independent researcher who was conducting the interviews also introduced themselves and explained the aims and purpose of the session.

Participants were asked about their reasons for participating in the BOB study and what they hoped to get out of the programme. At 3 months (half way through having the intra-gastric balloon in) and 12 months follow-up, 6 months post intra-gastric balloon removal, 3 months post lifestyle intervention) [9, 10] participants were asked to recount their early experiences of participating in the BOB study. Participants were also asked to identify key people who had supported them in their journey so far and how they had helped.

### Qualitative analysis

Framework analysis was used to analyse the data. Framework analysis was deemed an appropriate approach to analyse qualitative data due to the systematic nature of the approach [15]. The lead researcher firstly identified appropriate themes and associated sub themes that emerged from the data, at 3 months and then repeated for 12 months. Charts were then laid out on a thematic basis, which allowed identification of patterns, differences and similarities at each time point. Finally, diagrams were used to aid the final interpretation phase of the analysis. To counter bias and ensure the trustworthiness of the data, peer consultation took place between the researcher and two other colleagues on the development of the thematic framework, charting and mapping data, with final interpretations agreed.

## Results

This qualitative study aimed to provide in-depth experiences of participating in the BOB programme [9, 10] from the adolescent and family perspective. A full list of quotes can be found in Table 1.

**Table 1:** Complete list of BOB participant and family quotes – to be included at the end of the manuscript

Quote number	BOB ID	Age (years)	Gender	*Quote*
1	BOB 8	15	F	*“I wanted to lose weight and feel better in myself because right now I’m not feeling too good”*
2	BOB 9	14	M	*“I really wanted to improve my fitness because I thought I was really unfit and I was really fat. So I just wanted to obviously get a bit slimmer and get my fitness up”*
3	BOB 1	16	F	*“I’ve been overweight since I was little. I’ve tried tablets, I went to Shine. Nothing really worked. It was like doctors always said your resort’s surgery but I didn’t want permanent surgery”*
4	BOB 11	15	F	*At first I was very nervous, but then I read in to it. I was nervous about dying in the operation”*
5	BOB 7	16	F	*“It’s to be honest I don’t really notice it a lot, but most of the time it’s – it don’t really do much for me, like when I’m – I get weird like feelings in my stomach. Like when you, like when you, you can’t lay on your stomach on it because it feels like there’s a balloon in your stomach obviously, but you get random stomach rumblings, other than that I don’t not feel anything else*
6	BOB 3	15	F	*“I felt sick for quite a while, for like a week. But it felt quite weird because I felt like if I had a sandwich normally before I would have wanted something else. But if I just had that sandwich then I’m quite full and I didn’t really want anything else which seemed quite weird”*
7	BOB 4	15	M	*Well before I would normally skip breakfast. Then I’d have something at school, I’d probably have toast or a sausage sandwich. Then I’d have dinner at school but a main meal like fish and chips, or just some chips from the chip shop. Then I’d get home and I’d have like another sausage sandwich, then another big meal my mum cooked. And then I’d have crisps and chocolate and different sweets and lots of fizzy drinks. Now I’d have Weetabix with semi-skimmed milk and then an apple at 11. I have my dinner at one which would be pasta or sandwich then when I get home I’d have whatever my mum had cooked. The other night I had quiche and salad*
8	BOB 4	15	M	*“I think it’s the fact that it’s in there and you can feel it when you are eating. It makes you actually notice it, so you think about what you’re doing and whether you’re eating or whatever you take note of it. Not before you’re just eating and you don’t think about it”*
9	BOB 6	15	F	*“I was a bit disappointed because I had actually started trying. When you do these weight loss things and you give up halfway through, but then I actually started trying really hard and I put it back on, and I got a bit upset about it”*
10	BOB 3	15	M	*“it’s made a difference, if I keep working on it then it will be a bigger difference”*
				*“More weight loss, because you can get the ball rolling but it should just carry on”*
11	BOB 4	16	M	*Before the balloon was in I was just bad. I was putting on weight like two pounds every month and it as making me depressed which caused me to eat more. I wasn’t in an amazing place but going through the whole BOB trial its just makes me think about if I hadn’t done this what size would be now? Enormous”*
12	BOB 1	15	F	*“I’ve spent so many years trying to do something, looking abroad at surgery and if it wasn’t for this research then I wouldn’t be in the position I am now”*
13	BOB 1	17	F	*“Like I’d actually got really settled in and everything were going really really well and I were coming to the gym and doing all that lots but then it were time to come. And of course with other stuff going on in my life and it just too far to keep coming here*. *I know I’d asked about a gym membership”*
14	BOB 7	16	F	***“I lost it weight when it was in. yeah it gave me a kick start. But since the balloons out I put it back on.****I’m back to less exercise and probably eating more of the wrong stuff”*
15	BOB 8	15	F	*“it’s psychological you know the balloons in so you know you cant eat more as it will make you feel sick or whatever”*
16	BOB 3	16	M	*“It’s helped me mentally realise that this is not a quick fix and that I have to do it myself instead of looking for another way out. I realised that it had come out and then it was a like OK I need to get my arse into gear now”*
17	BOB 7	17	M	*“The feeling you get from being here and doing, and we talk and we laugh and we find, you know bits. It’s not like coming, its not a chore, it becomes, you want to come; its like coming to see a friend”*
18	BOB 6	15	F	*“Don’t even talk to me about friends. Don’t even talk about them. I don’t know who my friends are. Yeah but the only people I have at school now is Georgia Annabel Antonia and Page and its like they don’t even want to be my friends anymore. Because they don’t talk to me. I’m like the third wheel with them. It hard, it like it can’t speak to them about it though because they get all defensive and say it’s my fault. It just always puts me down”*
19	BOB 10	F	17	*“I thought well there’s nowt telling me I can’t eat, so can eat as much as I want now. So I’ll just go back to as I were type of thing”*
20	BOB 2	16	M	*“If you don’t work with the balloon, then it won’t work”*
21	BOB 3	16	M	*“I thought it would be a lot more easier, I just thought the balloon would do it all for me. But I’ve realised that wasn’t the answer, it was just the kick start to keep things going but it was just partly to help me but I had to do the rest myself’*
22	BOB 4	17	M	*“Its just not necessary to eat so much, there’s just no need for it. Maybe I used to use it as a way of coping emotionally but because I am now a lot happier that I’m not that weight I don’t snack because of it”*
23	BOB 9	M	14	*“I’m doing more, I’m actually walking to school. I’m walking a lot more, doing a lot more, eating less, changing my food and what I’m eating”*
24	BOB 12	F	13	*“I have some very supportive friends. My friends are always telling me to come out and we’ll go for a walk and stuff like that. And they’re walking the dog an’ stuff”*
25	BOB 12	F	13	*“I don’t know, like some people answer like oh yeah we’ll go out and do something and then other people would be oh yeah we’ll get a KFC or something stupid like that and you’re always battling between them*
26	BOB 6	F	14	*“The positives outweigh the negatives definitely. Well, when people brought me here a lot like week in and week out, and obviously mum brought me”*
27	BOB 4	M	15	*“My mum’s helped me a lot because like if I say oh don’t bring that into the house she’ll stop bringing it in the house. So if I ask her to get me some more water she’ll go shopping and get me some more water”*
28	BOB 1	F	16	*“I couldn’t do it on my own because well I live with my mum and brother. If they were against it or anything like that I couldn’t do it on my own. They’re there to support me”*
29	BOB 3	M	15	*“I just think when they’re giving advice and like when, they basically tell you how it is. Because I wouldn’t want them like tiptoe around me and just be easy, but when they just tell you how it is”.*
30	BOB 7	F	16	*“When she eats its like she’s having a snack. She’ll think about what she’s having but it’s also not as much as before. Whereas before it would have been a sandwich followed by a yogurt, followed by a chocolate bar followed by a biscuit followed by, she’d still be looking for something else, whereas now it could be yogurt and a biscuit or a piece of fruit and a biscuit”*
31	BOB 8	F	16	*“You learn like some stuff you can eat and you’ll be fine, and other stuff you can’t eat and you’ll get horrible bellyache. Like hash browns, I thought I’d be alright and I weren’t. I had McDonalds, I had chips and chicken nuggets and just like after a month of having it done I was in pain for 4 days. You learn what you can and can’t have”*
				*“That they believe that I can do it; like they support me, I support them and we support each other and they help, which is nice as some people don’t”*
32	BOB 6	F	15	*“I was a bit disappointed because I had actually started trying. When you do these weight loss things and you give up halfway through, but then I actually started trying really hard and I put it back on, and I got a bit upset about it”*
33	BOB 8	F	15	*“I eat less, because it makes you not feel hungry but I now eat healthier with more fruit and stuff. I still have to make sure I don’t eat too much, say if I ate now like I used to before then I would I would weight on”*
34	BOB 7	M	16	*“I mean I eat less but it [intra-gastric balloon] hasn’t worked as well as I thought it would because I still eat quite a bit of amount, its sort of a learning curve and to saying, I have to do it, not the balloon. It’s helped me more mentally instead of physically because I haven’t done a right deal of weight loss”*
***Parents of BOB participants***				
35	Mother BOB 2	N/A	N/A	*“We’ve come and done all the gym sessions haven’t we, I mean I started in a wheelchair as I’ve fluid on the brain and I was ever so stuffed. There were only little bits I could do, like the leg push down, the first time I couldn’t do it and was just hoisting, I couldn’t move I couldn’t do it. Today I’ve done it, put my legs up and its totally different. My movements totally different, everything different”*
36	Mother BOB 9	N/A	N/A	*“Obviously we do try and support him definitely. So obviously we do try and have meals that fit around what he [BOB participant] should have, that sort of thing. But we work silly hours me and his Dad so obviously our eating habits are different”*
37	Mother BOB 2	N/A	N/A	*“We all now eat low fat and skimmed milk, low fat spread, turkey rashers instead of bacon. Everything’s changed hasn’t it? We have Quorn meat instead – you know we are always looking for alternatives. Everything’s fresh and like we’ll sit and make our own kebabs but we do it together*
38	Mother BOB 2	N/A	N/A	*“I don’t know but when it came out something just clicked, just changed and he thought no, I got to do this for myself. We’ve changed everything what we eat. We try to eat the same as what [BOB] does, so that nobody’s having anything different”*
39	Mother BOB 2	N/A	N/A	*“We were getting to the point of despair weren’t we, we had tried everything. I did look at a gastric band but I didn’t think that was quite the answer, although that would be the last resort, I knew he [BOB] was getting into this I’ll go somewhere and they will fix me mentally. I knew deep down he needed a balloon first and if that didn’t work then it would be a gastric band. There are other way around it and if you don’t understand and take ownership, even with a gastric band you need to manage calories…… so I wasn’t comfortable to start but its been more than I expected….. It’s gone beyond physical, if you do this the balloon will help you. Its gone beyond that to him realising well I’ve got to work at this otherwise it’s not going to happen. Otherwise after the balloon you go back to square one, and fortunately he hasn’t, so it’s been fantastic”*
40	Mother BOB 3	N/A	N/A	*A bit annoying at times. The actual balloon didn’t work as well as I thought it might be have done, he obviously didn’t lose as much weight as I thought he might have done. But all other aspects and everything put together its been fine, that what I wanted for him, to be more social, go out more and a bit more active and self-confident, and he has”*
41	Mother BOB 2	N/A	N/A	*“We’ve done all sorts haven’t we before, and BOB used to get really disheartened because he’d lose weight and then it would go back on again and the he’d exercise. Then he’d be weighed on different scales and it was really getting him down, he missed a good 2 years of school because he just didn’t want to be around people”*
42	Mother BOB 6	N/A	N/A	*“I’ve suffered all my life, it’s been hard, I don’t want that for my child, she deserves better.”*
43	Mother BOB 7	N/A	N/A	*“It was just the fact that you hear all these stories of people having these gastric whatever they are and stuff like that, and they lose all this weight. So I’ll try it sort of thing because it should work. And it has up to now”*

### Perception of the intervention (reliance on balloon, “its down to me”)

All of the BOB participants had exhausted all available treatment options resulting in the perception that this study was a final opportunity, prior to considering bariatric surgery. Many alluded to feelings of desperation, following a history of failed weight loss attempts, and a sense of hope that this study would be effective in helping them lose weight. There was, initially, an overwhelming reliance on the intra-gastric balloon, with the majority of adolescents admitting that they believed the intra-gastric balloon would lose weight for them in the 6 months it was inserted. The adolescents believed that it would tell them when to stop eating therefore making weight loss much easier. Once the negative side effects had subsided after several days, many were surprised at how little they could feel of the intra-gastric balloon and how ‘normal’ they felt. At 12-months there was an apparent realisation by the group that they had to work with the intra-gastric balloon in order to optimise weight loss.

*“I thought it would be a lot more easier, I just thought the balloon would do it all for me. But I’ve realised that wasn’t the answer, it was just the kick start to keep things going but it was just partly to help me but I had to do the rest myself’ [BOB participant 3; Male 16 years]*

*“I mean I eat less but it [intra-gastric balloon] hasn’t worked as well as I thought it would because I still eat quite a bit of amount, its sort of a learning curve and to saying, I have to do it, not the balloon. It’s helped me more mentally instead of physically because I haven’t done a right deal of weight loss” [BOB participant 7; Male 16 years]*

Frequently, the families confused the study with bariatric surgery resulting in unrealistic expectations regarding the expected degree of weight loss in the 6-month period. Unrealistic goals and treatment expectations have been identified as a characteristic amongst adults seeking obesity treatment [16], yet whether this is consistent with obese adolescents is unknown. What was found, was that considering the perceived criterion for evaluating treatment success was weight loss; many of the participants saw the process as disappointing.

*“I was a bit disappointed because I had actually started trying. When you do these weight loss things and you give up halfway through, but then I actually started trying really hard and I put it back on, and I got a bit upset about it” [BOB participant 6; Female 15 years]*

### Longer-term strategies

Adolescents and parents perceived support ceased when the intra-gastric balloon was removed at 6-months, with poor attendance at the ‘maintenance phase’ of the study. When questioned during the focus groups, all participants emphasised a need for further support post-balloon removal. Previous research exploring the needs of obese adolescents identified ambivalence between personal motivations and their motives for treatment [16] highlighting a potential characteristic of this adolescent age group. That said, effective behaviour change strategies that prevent weight re-gain in severely obese adolescents post interventions are needed.

*“It’s helped me mentally realise that this is not a quick fix and that I have to do it myself instead of looking for another way out. I realised that it had come out and then it was a like OK I need to get my arse into gear now” [BOB participant 3; Male 16 years]*

### Environmental barriers

Multidisciplinary interventions that incorporate families into treatment are considered the ‘gold standard’ for treating obesity in children and adolescents [1, 5] yet there is limited research on how to engage family members within treatment in order to optimise outcomes [17]. Despite all families actively seeking treatment this was not necessarily an indicator of family motivation during treatment, a finding consistent with previous research [18]. Involvement and attendance of family members within the structured sessions differed significantly. One family consistently attended together, with all family members taking an active role in discussions and physical activity sessions, one adolescent forced her mother to stay in the car, whilst others attended with their family, but the attention was predominantly on the adolescents behaviour. The adolescent whose whole family attended did in fact lose the most weight, yet weight outcomes were variable and further research is needed to determine the specific role families play within treatment with severely obese adolescents.

*“The feeling you get from being here and doing, and we talk, and we laugh and we find, you know bits. It’s not like coming, its not a chore, it becomes, you want to come; its like coming to see a friend” [BOB participant 7; Male 17 years]*

All families raised similar barriers to attending the intervention, including travel time and/or distance to travel, along with the lack of time to commit to the weekly lifestyle sessions. When consideration was given to the implementation of behavioral change, all participants referred to feeling overwhelmed at implementing change within their chaotic daily lives. For example, this included parents juggling shift patterns at work, a perceived lack of control over their son or daughter’s behavior, financial worries and a perceived lack of time. One of the participants talked about caring for a disabled mother or looking after siblings, spending nights of the week between the mother and father’s house, with significant school and relationship issues with friends and family. Not surprisingly, this affected their ability to successfully implement their lifestyle goals.

*“Like I’d actually got really settled in and everything were going really really well, and I were coming to the gym and doing all that lots but then it were time to come out. And of course with other stuff going on in my life, it just too far to keep coming here . I know I’d asked about a gym membership..” [BOB participant 1; Female 17 years]*

Parental behaviours were also important influencing factors, something that was self-reported by parents and observed by the adolescent. These are associated with frequent physical activity [19] which not only predicted adolescent behaviour but also reduced BMI [20]. Verbal pressure from parents, such as restriction of certain foods, or verbal messages to eat less and exercise more - an observation within the interviews conducted here, has been associated with weight gain [20]. In this pilot BOB study [10] in four cases of weight gain at 12 months, some form of verbal pressure was identified or perceived by the young person during the qualitative interview. In comparison to BOB participants who lost weight at 12 months had full engagement from the family members with all family members making lifestyle changes too, evidenced within these qualitative findings This suggests that perceived parental modelling, and not perceived parental social pressure, could be an effective way of helping adolescents to reduce their weight.

*“We all now eat low fat and skimmed milk, low fat spread, turkey rashers instead of bacon. Everything’s changed hasn’t it?...... We’ve come and done all the gym sessions haven’t we, I mean I started in a wheelchair as I’ve fluid on the brain and I was ever so stuffed. There were only little bits I could do, like the leg push down, the first time I couldn’t do it and was just hoisting, I couldn’t move I couldn’t do it. Today I’ve done it, put my legs up and its totally different. My movements totally different, everything different” [Mother BOB participant 2]*

Reasons for not participating in the interviews included; not wishing to be audio recorded (*n* = 2); failure to attend appointment (*n* = 1) and an adverse event (unrelated to study delivery) resulting in a hospital admission (*n* = 1).

## Discussion

The aim of this qualitative study was to explore the experiences of the BOB intervention, intra-gastric balloon and lifestyle programme, from a participant and family perspective during and post-treatment.

### Key findings

At 3 months into the programme, participants discussed their decision-making about the programme which focused on wanting to fit in with peers and social networks, seeking improved confidence, health benefits and an opportunity to receive treatment prior to undertaking bariatric surgery. All (*n* = 11) had clear expectations of what they wanted from the programme including; increased confidence, weight loss, and to avoid bullying alongside the opportunity to shop for new clothes on the high street. The initial negative side effects of the intra-gastric balloon were discussed, which for some (*n* = 3) lasted longer than anticipated, causing distress from pain and sickness and diarrhoea (*n* = 1). For others (*n* = 5) this early phase was already associated with positive changes including an increased awareness of food choice, regularly eating breakfast, increased physical activity levels along with a sense of commitment to maintain this momentum for further change. In contrast, others (*n* = 3) expressed a sense of frustration, as weight loss was slower than expected. A common overwhelming theme in the interviews centred on the reliance on the intra-gastric balloon and that it would make weight loss easier from both adolescents and parents. Whilst interviews offered an insight into the habits of parents the accounts inferred a complex process within the wider family dynamic that requires additional exploration to understand how to facilitate a whole family approach to change. That said parental accounts identified an awareness of the impact of their behaviours on their children albeit there was limited evidence of their implementation of behaviour change. Distance to travel for the study and study logistics such as car parking, were discussed as initial barriers to engaging with the research study.

The 12-month interviews gave the opportunity for adolescents and their families to reflect on the overall BOB lifestyle programme experience. Perceived effectiveness of the programme was strongly influenced by the degree of weight loss experienced. Families who perceived the study a quick fix and relied on the intra-gastric balloon felt disappointed with weight loss. The majority however acknowledged that it had been a learning opportunity.

A consistent view held by all the participants and their families was the need for longer-term support post intra-gastric balloon removal, to encourage the maintenance of lifestyle behaviours. This is despite being offered a two-month maintenance period which none of the families attended consistently. Further research to understand the needs of adolescents and their families longer-term are needed. Parents empathised with the challenge of weight loss experienced by their children, yet the level of engagement observed from parents, in terms of attending the programmes and making lifestyle changes themselves, differed amongst the group. This reinforces the importance, albeit complex, of integrating the family and home environment within the delivery of obesity treatment for severely obese adolescents.

Qualitative research can provide a more in-depth understanding of participant perceptions, identifying what is important from a participant perspective [13]. This is particularly important in adolescents, specifically severely obese adolescents, where systematic review evidence on how to effectively manage obese adolescents is scarce [1]. This study aimed to bridge the gap in literature by providing in-depth experiences of severe obese adolescents who were involved in a novel treatment program specifically designed and tailored to the needs of this discrete and distinct population group. Adolescents described a clear desire for professional support in weight management, with many wanting continued support beyond the end of the formal BOB protocol. The critical role external support has in weight management amongst adults and children is well known yet there continues to be an incongruence between the experiences of participants and the essential program content delivered in weight management interventions [14]. Here, the integration of the home environment was well accepted amongst the severely obese families, but further work is needed to understand how this integration can be used to foster an environment conducive to the maintenance of positive lifestyle change [21]. Findings here support the integration of a gradual reduction in formalised support embedded within weight management design protocols [21] but with inconsistencies in what adolescents say they want and what they adhere to, a precise recommendation on program dose, is challenging. A clearer reporting of protocol design would be a start [22].

Findings here reiterate the need for whole families to be actively involved in behaviour change attempts highlighted by NICE guidelines [3].The engagement of families within the BOB intervention was positive from the adolescent’s perspective, aligning with existing research where family support was particularly helpful when they joined in with behaviour change efforts [13]. That said, the specific role families played within treatment appears complex, particularly in adolescents that reported limited success in weight loss or maintenance [13]. Research is needed to gain insights into adult caregiver’s views towards their role/ responsibilities in childhood obesity interventions [23]. Incongruence did exist between parents and adolescents when asked about their primary motivations for engaging in the study. Frequently, parents spoke of health concerns regarding their child, whilst adolescents focused on the desire to be socially accepted. As outlined by Daniellsson et al. 2015, adolescents interviewed here perceived healthy weight as being normal, with a belief weight loss being critical in being socially accepted, and a reduction in bullying [24].

The family and home environment is a highly influential psychosocial antecedent of adolescent obesity but less than a quarter of interventions include home visits [25]. Based upon this, the BOB formally integrated the home environment within the protocol design [24, 26] which although being positively reviewed by participants, the true value of its inclusion was beyond the scope of this pilot study. Additional research is therefore needed to assess its value, its role in long term maintenance as well as specific practical guidance for practitioners on how to embed within multi component programs.

### Strengths and limitations

There are limitations and advantages to our approach. The open-ended nature of this qualitative approach means it is possible that the researcher’s own views, conflicts and prejudices might have influenced the themes that were subsequently identified. The close rapport established between the lead researcher and the families could also have influenced the responses. On reflection, the independent researcher conducting the 3-month interviews affected the depth and quality of the interviews due to a perceived lack of rapport or trust from study participants. To resolve this, the lead researcher conducted the 12-month interviews. Having parents and families present during the interviews could have also affected the responses given by the adolescents. Therefore, an alternative method of collecting the qualitative data could have been implemented along with adolescent only interviews.

## Conclusion

This paper contributes to the limited evidence base of the lived experience of severely obese adolescents and their families participating in obesity treatments. The process of weight loss for severely obese adolescents appears complex. Treatment programmes must be system wide, supporting individual behavior change through behavior change techniques and supportive physical, social and policy environments. Whilst the extent to which family inclusion in treatment influences effectiveness remains unknown [27], it appears the home-based environment and the role of parent and families own lifestyle behaviors must be considered when designing adolescent treatment programmes. Further qualitative work should be conducted from the participant perspective to improve the effectiveness of obesity treatment programmes for severely obese adolescents and their families.

## Data Availability

The datasets used and/or analysed during the current study are available from the corresponding author on reasonable request. Data from the broader BOB study can be found here: Reece LJ, Sachdev P, Copeland RJ, Thomson M, Wales JK, Wright NP. Intra-gastric balloon as an adjunct to lifestyle support in severely obese adolescents; impact on weight, physical activity, cardiorespiratory fitness and psychosocial well-being. International journal of obesity. 2017 Apr;41(4):591-7.
